# Newly Designed Fluorescence In Situ Hybridization Probes Reveal Previously Unknown Endophytic Abilities of *Tuber magnatum* in Herbaceous Plants

**DOI:** 10.1007/s00248-025-02542-z

**Published:** 2025-05-08

**Authors:** Simone Graziosi, Lara Deloche, Mélanie Januario, Marc-André Selosse, Aurélie Deveau, Cyrille Bach, Zhixiao Chen, Claude Murat, Mirco Iotti, Philippe Rech, Alessandra Zambonelli

**Affiliations:** 1 Department of Agricultural and Food Sciences, University of Bologna, Viale G. Fanin 44, 40127 Bologna, Italy; 2 ISYEB, Muséum National d’Histoire Naturelle, CNRS, EPHE-PSL, Sorbonne Université, 57 Rue Cuvier, CP39, 75005 Paris, France; 3 Department of Plant Taxonomy and Nature Conservation, University of Gdańsk, Ul. Wita Stwosza 59, 80-308 Gdańsk, Poland; 4 Institut Universitaire de France, Paris, France; 5 Université de Lorraine, INRAE, 54000 Nancy, IAM France; 6 Department of Life, Health and Environmental Science, University of L’Aquila, Via Vetoio, 67100 Coppito, L’Aquila Italy

**Keywords:** FISH, Endophytism, Non-ectomycorrhizal host plants, Truffle ecology

## Abstract

*Tuber magnatum* Picco (the Italian white truffle) is the most valuable and widely appreciated truffle. It is an ectomycorrhizal fungus known to associate with many broadleaf tree species. However, its mycorrhizae are rarely observed in the field, suggesting possible alternative symbiotic strategies, such as endophytism with non-ectomycorrhizal plants. In order to test potential endophytic interactions of *T. magnatum* with wild plants, a combination of polymerase chain reaction (PCR) and Fluorescence In Situ Hybridization (FISH) approaches were used. Specific FISH probes for *T. magnatum* were designed, tested in vitro on hyphae and/or ectomycorrhizae, and selected for their specificity. These probes were then used on a wide variety root samples of wild plants collected from three *T. magnatum* production areas in Italy and previously tested for the presence of *T. magnatum* mycelium using PCR-specific primers. Molecular analyses detected the presence of *T. magnatum* in 21 of 100 plant samples analyzed. FISH analysis confirmed the extracellular presence of active *T. magnatum* hyphae inside the root system of *Carex pendula* Huds plant. This study provides the first evidence of *T. magnatum* acting as an endophyte in an herbaceous plant. The newly designed, highly specific *T. magnatum* FISH probes can be used for further investigations to confirm the endophytic tendencies of *T. magnatum* and to understand their influence on the life cycle and biology of this fungus.

**Supplementary Information:**

The online version contains supplementary material available at 10.1007/s00248-025-02542-z.

## Introduction

Among many fungi fruiting hypogeously, true truffles refer to all fungi belonging to the genus *Tuber* within the family Pezizaceae, phylum Ascomycota [1, 2]. Approximately 180–220 species have been estimated in the genus [3], but only a few produce ascomata with significant gastronomic and economic value: the European species *Tuber magnatum* Picco (the Italian white truffle), *Tuber melanosporum* Vittad. (the Perigord black truffle), *Tuber aestivum* Vittad. (the Summer truffle), and *Tuber borchii* Vittad. (the Bianchetto truffle) are the most sought-after, with prices ranging from €30 to €6,000 per kilogram, depending on the species, size of the ascomata, season, ripeness, and availability. Among these, *T. magnatum* stands out for its intense aroma and unique flavor, making it the most prized truffle. Consequently, retail prices for *T. magnatum* in Italy are higher than for any other truffle, placing it among the world’s most expensive foods [4].

*Tuber magnatum* is an ectomycorrhizal fungus known to associate with many broadleaf tree species, particularly *Populus* spp., *Quercus* spp., and *Tilia* spp. [5]. In Italy, numerous cultivation attempts using inoculated plants from nurseries were undertaken from the late 1990 s, yielding variable and sporadic results [4, 6]. Notably, all these plantations were established in areas where *T. magnatum* naturally occurred. Considering this, it cannot be ruled out that production in the early *T. magnatum* plantations was a spontaneous event resulting from secondary local inoculation, rather than a direct result from planting inoculated plants. Only recently, a *T. magnatum* orchard established in France outside its natural range in 2015 [7] yielded the first ascoma production [8]. This undoubtedly confirmed the possibility of cultivating this *Tuber* species by planting trees that had been previously inoculated in greenhouses.

Interestingly, *T. magnatum* mycorrhizae can be obtained under greenhouse conditions [9–11], but they are rarely found in the field, even in productive places [12, 13]. Despite the rarity of its mycorrhizae, *T. magnatum* mycelium is consistently present and extensively colonizes the soil in the productive areas [14, 15]. The ectomycorrhizal habit of *T. magnatum* is also suggested by its limited capacity for saprotrophic metabolism of its genome and the challenges in isolating its mycelium in vitro [4, 16], characteristics common among many mycorrhizal fungi [17–19].

Compared to mycorrhizal fungi, endophytes produce no clear external morphological features, are often less abundant in plant tissues, and do not strongly contribute to host nutrition [20]. Recently, other *Tuber* species such as *T. melanosporum*, *T. aestivum*, and *T. borchii* have been shown to develop as endophyte within non-ectomycorrhizal plants including orchids [21–27]. Their role in *Tuber* biology is still not clear, but some authors speculated that orchids and other herbaceous species could be involved in *Tuber* spp. life cycle acting like reservoir of the paternal genotype [28, 29] although only maternal genotype was found in herbaceous roots to date [26]. Taschen et al. [30] documented that *T. melanosporum* developed more efficiently (with higher mycelial abundance) in rhizotrons containing non-ectomycorrhizal plants, with negative effects on the growth of these plants. Other authors found opposite results with a decrease of *T. melanosporum* mycelium and ectomycorrhizae in a dual cultivation of truffle mycorrhized plants with herbaceous or aromatic plants [31, 32]. In the field, *T. melanosporum* mycelium was not statistically affected by the presence of aromatic plants [33]. Our current knowledge of the interaction between truffles and these non-ectomycorrhizal plants is limited, and it is not known whether or not such endophytism is important in the life cycle of truffles. It cannot be excluded a sort of nutritional exchange between these plants and the *Tuber* mycelium, including carbon extraction from *Tuber* spp. by orchids [34] or providing of N to the plant [26]. However, it is a challenging task to distinguish the carbon flux originating from ectomycorrhizal plants, which is typically large and consistent [35–37], from that of non-ectomycorrhizal endophyted hosts where it varies widely among species [38], as both types of hosts are simultaneously colonized by truffle mycelium.

Most of these studies relied on polymerase chain reaction (PCR) or next-generation sequencing (NGS) methods to identify *Tuber* endophytisms. Notably, *T. melanosporum* endophytism was confirmed in one study through Fluorescence In Situ Hybridization (FISH) targeting cytoplasmic ribosomes, which demonstrated endophytism within the roots and excluded simple rhizoplane colonization [26]. FISH is a valuable tool for investigating the spatial distribution and interactions of microorganisms within complex environmental samples. By utilizing nucleic acid probes specific to ribosomal RNA, FISH enables direct visualization of living microorganisms, including fungi, within their natural habitats, providing critical insights into their ecological roles and interactions [39–43]. Recent advancements, such as confocal laser scanning microscopy (CLSM) and multicolor FISH using extended fluorophore sets, have enhanced its ability to detect multiple microbial taxa simultaneously [44–47]. Applied to fungal communities, these methodologies can provide detailed insights into their spatial distribution in natural matrices beyond molecular evidence. Furthermore, optimized protocols for sample fixation and hybridization have improved the resolution and accuracy of bacterial and fungal detection in highly complex matrices like soils and rhizospheres [42, 48–51]. In fungal ecology, FISH is particularly valuable for elucidating the structure and function of ectomycorrhizal fungi. However, its application to label fungal hyphae directly has been limited. Instead, most studies so far have focused on fungal interactions with bacteria, providing insights into belowground ecosystem dynamics [52–54].

This study aims to investigate the potential interaction of *T. magnatum* mycelium with wild plants, mainly herbaceous, collected in the field, combining PCR and FISH approaches. Specifically, the objectives were (1) to collect ectomycorrhizal and non-ectomycorrhizal plants from *T. magnatum* production areas; (2) to detect molecularly the presence of *T. magnatum* mycelium in the soil and within the roots of these plants, using species-specific primers; (3) to design and select specific FISH probes targeting *T. magnatum* mycelium and ectomycorrhizae; and (4) to use the selected probes to confirm the presence of *T. magnatum* inside roots resulted positive through species-specific PCRs.

## Material and Methods

### Sites of Study

Three natural fruiting sites (Supplementary Table S1) were selected to investigate the potential endophytic behavior of *T. magnatum* with ectomycorrhizal and non-ectomycorrhizal host plants that share its habitat. The first site was a rural area near Città della Pieve (subsequently named CDP, Perugia, Umbria, Italy), at an elevation of approximately 430–455 m a.s.l. This hilly region is characterized by alternating scrubland and cultivated fields. The putative host plants in this area include *Corylus avellana* L., *Ostrya carpinifolia* Scop., *Populus nigra* L., *Populus alba* L., *Quercus cerris* L., *Quercus pubescens* Willd., *Quercus robur* L., and *Salix* spp. The second site, the Panfilia Forest (subsequently named PF), is an ancient lowland forest (30 m a.s.l) located between the provinces of Bologna and Ferrara, near Sant’Agostino (Ferrara, Emilia Romagna, Italy). The predominant putative host plants in this area include *C. avellana*, *P. alba*, *Q. robur*, *Salix* spp., and *Tilia cordata* Mill. The third site is an Apennine mountainous area near Montefalcone nel Sannio (subsequently named MNS, Campobasso, Molise, Italy) at 345 m a.s.l. This site consists of a mesophilic deciduous forest, where possible symbiotic partners include *C. avellana*, *O. carpinifolia*, *P. alba*, *Q. cerris*, *Q. pubescens*, and *Q. robur*. Additional characteristics of the sites (soil, average precipitation and temperatures) are reported in Supplementary Table S1.

### Ascomata, Soil, and Wild Plants Sampling

In January 2022, the study sites were visited with trained dogs in order to detect the *T. magnatum* production patches. The truffle hunters indicated *T. magnatum* fruiting points (FPs) based on previous harvests or picking up new ascomata for each site and their positions were georeferenced (GPS coordinates of each ascoma collection point were recorded but not showed at the request of truffle hunters). Six, three, and one FPs were respectively found for CDP, PF and MNS.

For each FP, nine equidistant soil cores were collected with 1.6 cm diameter disposable polyvinyl tubes along two diagonal lines in a 2 m^2^ area centered in FPs. The upper layer of organic matter was removed, and the soil was sampled down to a depth of 20 cm. The soil was maintained at 4 °C and extracted from the tubes within 24 h. Organic debris was removed, then the soil was sieved using 2-mm mesh and lyophilized. Successively, the soil was ground and stirred in a mortar. All the tools were washed and then sterilized with 5% bleach solution. The nine soil samples of each FP were mixed and the total DNA was extracted from three samples of 0.5 g taken from the composite sample according to Iotti et al. [55]. Then, the DNA was purified using the Nucleospin Plant II kit (Macherey–Nagel, Germany) and preserved at − 80 °C pending molecular analysis.

The mycelial presence in each selected FP was verified by amplification of the *T. magnatum* DNA in the soil with a nested PCR approach. The fungal DNA was first amplified with the fungal universal primers ITS1f/ITS4 [56, 57] (Supplementary Table S2), then the amplified fragments were subjected to a second PCR round by using the specific primers TmagI-TmagII [58] (Supplementary Table S2).

Since the Panfilia Forest is renowned as one of the most productive *T. magnatum* forests in northern Italy [59, 60], it was selected as representative fruiting site to investigate the most suitable season to detect root colonization by *T. magnatum* in ectomycorrhizal and non-ectomycorrhizal plants. Thus, in winter, spring, summer, and autumn 2022, for each FP where *T. magnatum* DNA was detected in soil samples, plant samples were collected from the nodes of a 1 × 1 m grid with an approximately 20-cm pitch placed on FPs (Fig. S1). The 10 plants closest to the FP were selected for each FP. The collected plants were identified, and the epigeal part was separated and preserved in the herbarium “Centro di Micologia” of Bologna (CMI-UNIBO). The roots were washed under tap water and analyzed on the same day. The same sampling procedure was applied in the other two fruiting sites in spring 2023, which was inferred as the most suitable season from the analysis of the PF root samples.

### PCR Detection of *T. magnatum* in Plant Roots and RNA Fixation Process

Three thin and healthy roots were selected from each plant sample and cut into three 1-cm fragments. The proximal and distal fragments were used for DNA extraction. They were superficially sterilized by immersion in 70% ethanol solution for 5 min, in 0.9% sodium hypochlorite solution for 15 s and then rinsed three times in sterile water following the protocol of Cao et al. [61]. This protocol was previously applied to detect the endophytic root colonization of *T. aestivum* and *T. melanosporum* in non-ectomycorrhizal plants [26]. To check for the presence of *T. magnatum* DNA inside the roots, total DNA extraction from proximal and distal fragments were completed as previously reported by Graziosi et al. [27] and nested PCRs were performed using specific primers for *T. magnatum* (TmagI-TmagII, [58]).

The 1 cm central fragment was put into a 4% paraformaldehyde in 1 × Phosphate Saline Buffer (PBS 1 ×), added with 0.1% of Tween20. All the solutions were prepared with Ultrapure Water, Milli-Q®. The fixing solution was stored on ice before sample immersion. Three successive vacuums were then applied. The last vacuum was maintained for 1 h at room temperature. Then the root pieces were rinsed three times in PBS 1 × for 10 min and immersed consecutively in ethanol 10%/PBS 1 × solution for 30 min, in ethanol 30%/PBS 1 × solution for 30 min and, at the end, in ethanol 50%/PBS1 × solution for 30 min. These last immersion steps were conducted at room temperature. The samples were preserved at − 20 °C pending FISH experiments.

### Design of rRNA Specific Probes for *T*. *magnatum* Involved into FISH and Microscopy Observations

The first step was the in silico design of new probes based on the alignment of SSU ribosomal DNA (rDNA) sequence of *T. magnatum* and other *Tuber* spp., alongside sequences from various species in the Pezizales and other orders obtained from genomic data published in GenBank (NCBI, [62]) and Mycocosm (The Fungal Genomics Resource-JGI, [63]) databases (Supplementary Table S3). SSU rDNA sequences were aligned using the Multalin algorithm [64] (Fig. S2) to find variable domains for developing *T. magnatum* species-specific or *Tuber* genus-specific probes.

Four regions exhibiting polymorphisms were retained as a basis for designing probes targeting *Tuber* spp. or *T. magnatum*, including closely related species in the Tuberaceae family. Probes were evaluated for their accessibility [65] and in silico specificity by using iterative BLAST searches with low stringency algorithm parameters in the GenBank and SILVA databases [66]. Finally, the number of mismatches of the designed probes, the in silico accessibility, and specificity were taken into account to choose four probes named T.mag185, T.mag645, T.mag1313, and T.mag1647, each presenting a different degree of specificity to *T. magnatum* (Supplementary Table S4). All FISH-probes were commercially synthesized by Biomers.net (Ulm/Donau, Germany) including 5′-end labeled with Atto fluorochromes and stored in sterile DNA-grade water at − 20 °C.

To highlight genetic diversity between SSU sequences of *T. magnatum* and other closely related fungal species, a phylogeny was inferred by the Maximum Likelihood (ML) option implemented in MEGA11 software [67]. ML analyses were performed with 1000 throughout bootstrap replicates (100 runs), applying the models of nucleotide substitution K2 + G + I (Fig. S3).

### Collection and Fixation of Mycelial Samples and Ectomycorrhizae

To evaluate the in vitro sensitivity and specificity of the FISH probes and to establish confocal acquisition parameters, strains of several fungal species were selected based on their genetic closeness to *T. magnatum* by assessing the similarity of their SSU rDNA sequences. Closely related *Tuber* spp. and outgroup species in the order Pezizales included in the analysis were: *T. aestivum* (strain 5230), *T. borchii* (strain IT1) *Tuber brumale* Vittad. (strain 5256), *T. melanosporum* (strain TME2, [68]), and outgroup species belonging to Pezizales order, *Morchella eximia* Boud. (strain MD14) and *Dissingia leucomelaena* (Pers.) K. Hansen & X.H. Wang (strain DILE1). Several of these fungal species were chosen also because they often share the same habitat of the white truffle, occasionally colonizing the roots of the same host tree [60].

All selected pure cultures were cultivated on M9 Minimal Medium (M9 MM, [69]) at 22.5 °C in the dark. Each pure culture, excepted *T. magnatum*, was previously isolated from ascomata by excising and aseptically transferring 1–2-mm fragments of fungal tissue from the inner part of the ascoma onto M9 MM. *Tuber magnatum* sample (herbarium number 5377) was taken from the gleba tissue of a young ascoma provided by Truffleland s.r.l (Sant’Anatolia di Narco, Perugia, Umbria, Italy). Only for *M. eximia*, a diluted spore suspension in sterile water was employed, added to M9 MM with antibiotics (200 μg/mL of streptomycin, ampicillin, and chloramphenicol). The ascomata were then dried and deposited in the herbarium of the “Centro di Micologia” of Bologna (CMI-UNIBO). Once fungal elements were discernible to the naked eye on the Petri dishes, agar plugs containing hyphae were harvested and fixed as previously reported and finally stored at − 20 °C in 50% ethanol-PBS 1 × until use. The same process was applied to *T. magnatum* gleba tissue pieces.

Moreover, *Tuber* spp. ectomycorrhizae from inoculated seedlings were included in this preliminary phase to evaluate the efficiency of the FISH probes on fungal hyphae interacting with plants in semi-natural conditions. Young *T. magnatum*, *T. aestivum*, and *T. melanosporum* ectomycorrhizae were individually sampled from *Q. pubescens* inoculated plants produced by Robin Pépinières (St Laurent du Cros, France). Each *T. magnatum* ectomycorrhiza was cut in half with a razor blade, and one half was immediately fixed in a 4% paraformaldehyde solution. After 4 h of fixation on ice, the ectomycorrhizae were washed three times in PBS 1 × solution, partially dehydrated in an ethanol series and then stored at − 20 °C in 50% ethanol/PBS 1 × until use. The other half was used for DNA isolation using Extract-N-Amp Tissue DNA extraction (Sigma, Merck KGaA, Germany). DNA extraction was performed according to manufacturer instructions. For *T. magnatum* ectomycorrhizae, the internal transcribed spacer (ITS) was amplified using universal fungal primers ITS1 F and ITS2 (Supplementary Table S2) [57], and sequenced by Eurofins (Ebersberg, Germany). BLASTn against the NCBI-nr database was used to confirm the species identity. The sequences were deposited in the GenBank under the following accession numbers: PQ818236 to PQ818245.

### Test of Newly Designed Probe *In Vitro* Specificity

The FISH experiment was conducted similarly to Laurent-Webb et al. [43]. Rapidly, samples of both ascomata and mycelium were gradually dehydrated with an ethanol series (50%, 70%, 96%, 100%) in ultra-pure water and then rehydrated with an ethanol series (70%, 50%, 30%, 10%) in PBS-T (1 × PBS with 0.1% Tween 20), for 15 min each, ending with three 10-min PBS-T baths. All steps occurred at room temperature.

Samples were treated with digestion solution (25,000 U/mL Lyticase, 12.5 U/mL Chitinase in PBS 1 ×) for 15 min at 30 °C to weaken fungal cell walls. A pre-hybridization buffer (30% formamide, 5 × SSC, 0.1% Tween 20, 100 μg/mL tRNA, 0.2 × Denhardt’s solution) was then added, followed by a 30-min incubation at 46 °C. The buffer was replaced with a hybridization solution containing probes (0.35 pmol/µL of hybridization solution each), incubated for 2 h at 46 °C. After a 10-min wash (0.2 M Tris HCl pH 8, 0.005 M EDTA pH 8, 0.7 M NaCl, 0.1% SDS) and PBS 1 × rinse, samples were mounted in Slowfade Diamond Antifadent (Molecular Probes, Eugene, OR, USA, Thermofisher) solution containing 0.1% SR2200, a cell wall dye (Renaissance Chemicals, North Duffield, UK) and visualized with a confocal microscope (Zeiss LSM980, Carl Zeiss, Oberkochen, Germany) coupled to ZEN Blue 3.3 software (Carl Zeiss, Oberkochen, Germany). The acquisitions were performed in confocal mode. Fungal samples were co-hybridized with the universal eukaryote probe Euk516 and the four designed probes individually or with an anti-sense, non-binding probe (AS-Euk516 or AS-Rus899), each tagged with distinct fluorescent dyes (Supplementary Table S4), or undertaken the hybridization process without any probe addition as a control. The probe AS-Rus899 is specific to *Russula* spp. and *Lactarius* spp. Atto565 and Atto633 probes were excited at 561 nm and 639 nm, respectively, and detected with bandpass filters (570–630 nm, 640–720 nm). The cell wall dye SR2200 was excited at 405 nm and detected with a 420–477 nm filter.

In these co-hybridization experiments, probe sensitivity and specificity to bind to the 18S RNA target were evaluated in comparison to the positive signal obtained with the eukaryotic probe Euk516 [70]. Fluorescence intensity signal obtained for each sense probe was also compared with negative hybridization controls, i.e., FISH experiments realized with AS-Euk516, AS-Rus899 probes, without probes or FISH realized with sense probes on other fungal species than *T. magnatum*. These initial steps were used to calibrate laser power and photodetector gain parameters while minimizing tissue autofluorescence. Moreover, FISH experiments were conducted to verify the specific binding of *T. magnatum* FISH probes outside of in vitro conditions, on fungus-plant tissue of ectomycorrhizae. The hybridization protocol was the same as that applied to ectomycorrhizal and non-ectomycorrhizal plant root samples, as reported in the paragraph: “Hybridization and observation of *T. magnatum* hyphae in roots of wild plants.” Along with *T. magnatum*, *T. aestivum* and *T. melanosporum* ectomycorrhizae were added as negative controls. For each *T. magnatum* specific probe, three sets of independent FISH experiments were performed and each time the signal originated from eight Z stacks were compared for both every fungal species and FISH experiment. The hybridization signals were obtained and analyzed by using the same parameters (laser power and gain of detector) and were also used to image the control samples: samples without any probe or samples hybridized with the anti-sense probes.

### Hybridization and Observation of *T.magnatum* Hyphae in Roots of Wild Plants

The *T. magnatum* specific T.mag1647 FISH probe was selected and used for FISH experiments applied on the roots from ectomycorrhizal and non-ectomycorrhizal host plants. FISH experiments on root samples of wild plants follow the same protocol, adapted from Laurent-Webb et al. [43]. Root fragments positive for *T. magnatum* through species-specific PCR were selected (TmagI-TmagII, [58], Supplementary Table S2). One PCR-negative root fragment for each of the PCR positive species was added to the experiment as control. Co-hybridization FISH experiments were done by using two probes targeting 18S rRNA simultaneously: the universal eukaryote probe Euk516 coupled with Atto-565 dye and newly designed T.mag1647 specific probe (Supplementary Table S4) coupled with Atto-633 dye. These fluorochromes were carefully chosen to reduce autofluorescence background of plant/fungal tissues [43].

Root samples were dehydrated using an ethanol series in ultrapure sterile water (50%, 70%, 96%, 100%) for 20 min per step at room temperature. They were immersed in pure methanol for 10 min at room temperature, refreshed, and stored at − 20 °C overnight. After two 5-min methanol washes at room temperature, the samples were rehydrated through a methanol series (70%, 50%, 30%, 10%) prepared in PBS-T, with each step lasting 20 min at room temperature. Samples were treated with a mix of plant and fungi cell-wall digestion cocktails [43] at 35 °C for 15 min to weaken cell walls, and rinsed three times in PBS-T at room temperature. They were then treated with 0.08 µg/µL of Proteinase K solution for 15 min at 37 °C. The reaction was stopped with 0.2% glycine prepared in PBS-T for 2 min, followed by a 30-min incubation in a post-fixative solution (4% paraformaldehyde in PBS-T). After three rinses in PBS-T, samples were prehybridized at 46 °C for 30 min, and then incubated with a hybridization buffer containing probes (0.35 pmol/µL) for 2 h at 46 °C. After a stringent 10-min wash at 46 °C in a buffer (0.2 M Tris–HCl pH 8, 0.005 M EDTA pH 8, 0.7 M NaCl, 0.1% SDS), samples were mounted in an antifade solution containing 0.1% SR2200 to visualize plant and fungi cell walls. Hybridized samples were stored at 4 °C until microscope observation.

Root samples were observed using a Zeiss LSM 980 confocal microscope equipped with EC-Plan Neofluar 10 ×/0.3 dry, W Plan-Apochromat 20 ×/1.0 water, and Plan-Apochromat 63 ×/1.4 oil objectives. Imaging employed the Airyscan 2 module in multiplex 4Y mode, with Atto565 and Atto633 probes excited at 561 nm and 639 nm, respectively, and detected with bandpass filters (570‒630 nm, 640‒720 nm). The cell wall dye SR2200 was excited at 405 nm and detected with a 420–477-nm filter. Fields of view were imaged with optical sections (Z-step: 0.15‒1 μm). Image analysis was conducted using ZEN Blue 3.5, ZEN 2.1 LITE (Zeiss), Vision4D 3.0.1 (Arivis AG, Germany), and FIJI-ImageJ softwares. The hybridization signals were obtained by using parameters (laser power and gain of detector) optimized during FISH experiments on pure fungi and were also used to image the control samples: samples without any probe or samples hybridized with the anti-sense probes.

## Results

### Endophytic Colonization Verification via PCR

The mycelium of *T. magnatum* was successfully detected in the soils of all FPs by PCR with *T. magnatum* species-specific primers. A total of 100 plant samples (60 from CDP, 30 from PF, and 10 from MNS; Table 1, Supplementary Table S5) were collected in the spring of 2022 and 2023 at fruiting points from the three fruiting sites. Most of the collected plants were non-ectomycorrhizal (97), while only three seedlings of ectomycorrhizal species were sampled (*C. avellana*, *P. alba*, and *T. cordata*).

**Table 1:** Results of species-specific PCRs for *Tuber magnatum* Picco on plant samples collected in this study in spring 2022 and 2023

Fruiting sites	Fruiting points (FPs)	Analyzed plant samples (analyzed species)	Positive plant samples	Percentage positive plant samples	Positive species^a^
Città della Pieve (PG)	6	60 (23)	15	25	*Arum italicum* {2}, *Sison amomum*, *Taraxacum dissectum*, *Galium verrucosum*, *Hedera helix* {3}, *Primula vulgaris* {3}, *Ranunculus bulbosus*, *Urtica dioica* {2}, *Viola odorata*
Panfilia Forest (FE)	3	30 (10)	5	17	*Acer campestre*, *Carex pendula* {4}
Montefalcone nel Sannio (CB)	1	10 (6)	1	10	*Ajuga reptans*
Total	7	100	21		

^a^Within brackets, numbers of plant samples with more than one positive sample

Among non-ectomycorrhizal plants, Asteraceae showed the highest species richness (Fig. S4), with seven taxa, followed by Apiaceae, Geraniaceae, Ranunculaceae, and Rosaceae with two species each. All other families exhibited just one species. In terms of samples, the families Arialaceae, Cyperaceae, and Asteraceae were the most abundant with 15, 12 and 12 plant samples, respectively. Primulaceae, Ranunculaceae, Araceae, Rosaceae, Geraniaceae, Violaceae, Urticaceae, Apiaceae, Plantaginaceae, and Lamiaceae showed a decreasing number of plant samples, from nine to two, while the other families accounted for just one plant sample.

The mycelium of *T. magnatum* was detected inside plant samples only in spring, while no sample from the other seasons (47% of the total plant samples) showed positive results. In detail, it was found in 21 out of the 100 examined plant samples, considering as positive the samples where at least one root gave specific amplicons (Table 1). All the positive samples were from non-ectomycorrhizal plants whereas the ectomycorrhizal plants gave no positive results. The highest number of positive plant samples were found in CDP with 15 samples (25% of total plant samples) followed by PF with five samples (17%) and MS with just one sample (10%).

The plant families with the highest number of positive samples were Cyperaceae (4), Araliaceae (3), Primulaceae (3), Araceae (2), and Urticaceae (2). In contrast, Apiaceae, Asteraceae, Lamiaceae, Ranunculaceae, Rubiaceae, Sapindaceae, and Violaceae yielded only one positive sample each. All other families provided no positive samples. See Supplementary Table S5 for a detailed report.

**Fig. 1:**
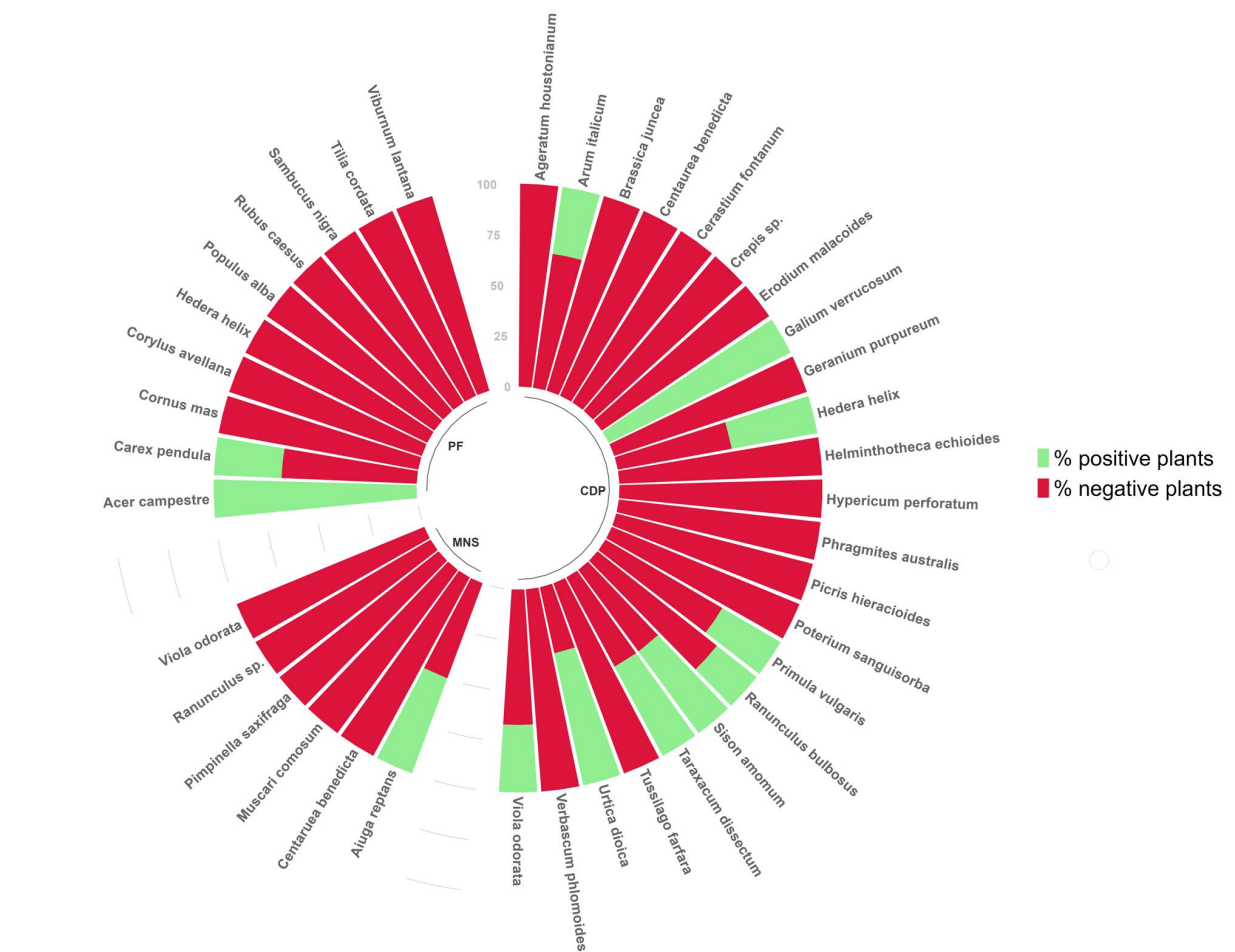
Percentual distribution of positive and negative plant samples collected in Spring 2022 and 2023 after PCR with *Tuber magnatum* Picco specific primers

Among the 39 examined plant species, 12 showed positive results (Table 1, Fig. 1). In details, *Acer campestre* L. and *Galium verrucosum* Huds. were represented by just one collected plant sample resulted positive by species-specific PCR (100%). Other species had just one positive plant sample, but more than one plant was collected: *Aiuga reptans* L. (2 plant samples in total, 50% positive plant samples), *Ranunculus bulbosus* L. (4, 25%), *Sison amomum* L. (2, 50%), *Taraxacum dissectum* (Ledeb.) Ledeb. (2, 50%), and *Viola odorata* L. (3, 33%). Two or more positive plant samples were detected in four species: *Arum italicum* Mill., (6 plant samples in total, 33% positive plant samples) *Carex pendula* Huds. (12, 33%), *Hedera helix* L. (7, 43%)*, P. vulgaris* Huds. (3*,* 33%), and *Urtica dioica* L. (2, 67%) (Fig. 1).

### Selection of Probes by *In Vitro* Pre-tests

The strategy of targeting 18S RNA for probes design is based on its high abundance in cells, which greatly enhances its detection. The design of the FISH probes was carefully tailored to achieve either a broad or narrow spectrum of specificity, to ensure the target recognition. The SSU sequences of *Tuber* species, including *T. magnatum*, exhibit an exceptionally high degree of identity, not only among themselves but also with closely related fungi such as *Choiromyces* spp. This high sequence conservation posed a significant challenge for probe design, as it left very few regions with polymorphisms suitable for differentiation. Consequently, the available regions for probe targeting were limited, influencing the specificity range of the probes. The designed probes demonstrated a specificity spectrum that was inherently constrained, being more or less selective for *T. magnatum*. In our design and based on in silico analysis, two probes were expected to be highly specific to *T. magnatum* (T.mag185 and T.mag1647), while probe T.mag1313 would include a subset of other *Tuber* species (such as *T. magnatum*, *T. borchii*, and *T. puberulum*) and probe T.mag645 should recognize almost all *Tuber* spp. as well as some *Choiromyces* spp. (Fig. S2).

**Fig. 2:**
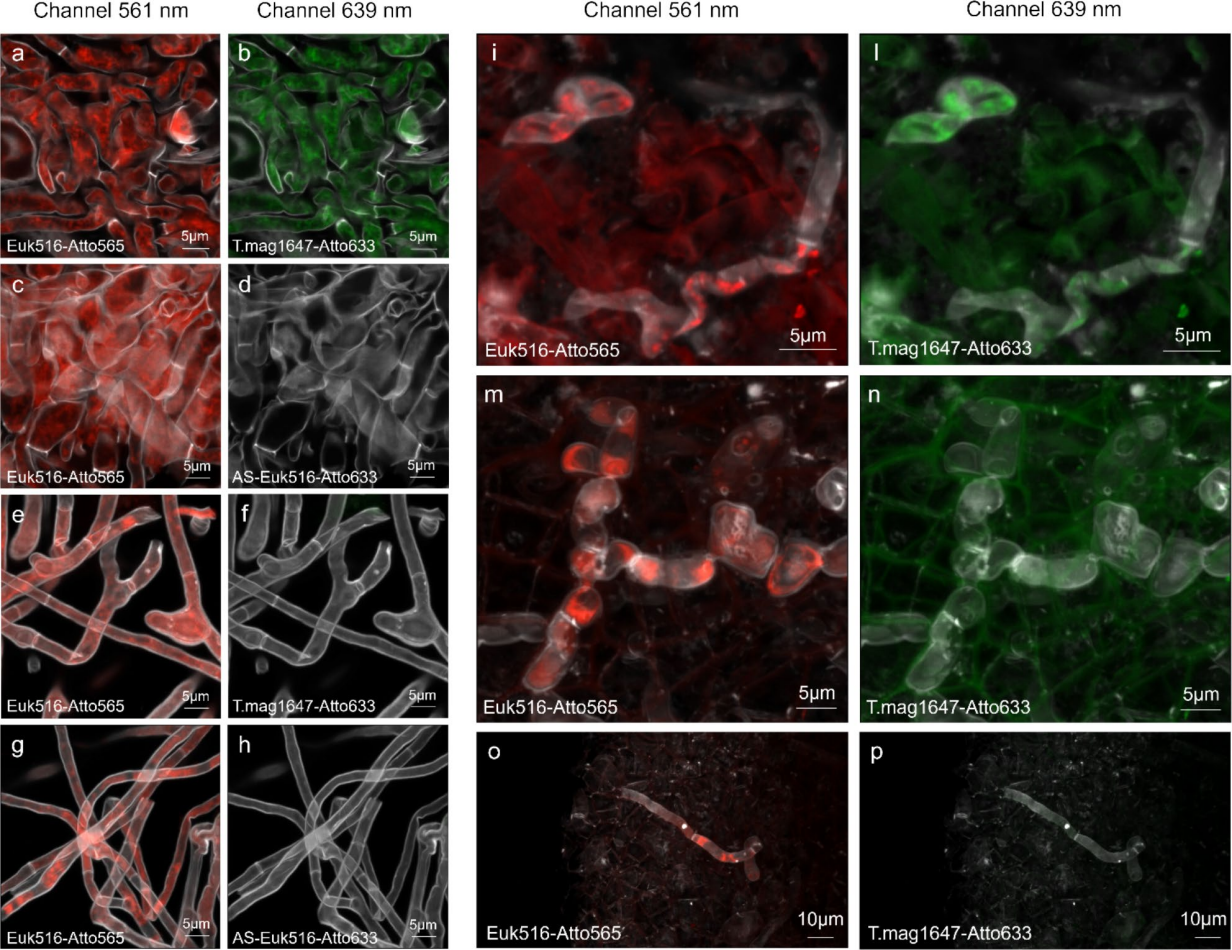
Evaluation of the Fluorescence In Situ Hybridization (FISH) probe T.mag1647, targeting the 18S ribosomal RNA of *Tuber magnatum* Picco, was conducted using either ascomata or pure cultures. In the figure is showed the comparison between the ascoma of *T. magnatum* (**a**–**d**, left panel), a pure culture of *Tuber melanosporum* Vittad. (**e**–**h**, left panel), and ectomycorrhizae of *T. magnatum* (**i**, **l**), *Tuber aestivum* Vittad. (**m**, **n**), and *T. melanosporum* (**o**, **p**) (right panel). The co-hybridizations were performed on *T. magnatum* ascoma (herbarium number 5377), *T. aestivum* (strain 5230), and *T. melanosporum* (strain TME2). The mycorrhizae were provided by Robin Pépinières nurseries (St. Laurent du Cros, France). Co-hybridization experiments were performed with a generalist probe targeting eukaryotic cells, Euk516 (in red), as a positive control. The positive control showed high signal intensity in the cytoplasm of fungal cells (**a**, **c**, **e**, **g**, **i**, **m**, and **o**), in combination with the T.mag1647-specific probe (**b**, **d**, **f**, **h**, **l**, **n**, and **p**). An anti-sense probe, AS-Euk516, was used as a negative control to verify the absence of non-specific binding of the probe or random fluorochrome binding. For each co-detection, 2D images from individual channels are presented. Fluorochrome Atto565 and Atto633 were excited at 561 nm and 639 nm, respectively, and detected with bandpass filters (570‒630 nm, 640‒720 nm). The cell wall dye SR2200 was excited at 405 nm and detected with a 420–477-nm filter

During our pre-test of FISH probes, all the four newly designed probes (Supplementary Table S4) showed a positive binding with *T. magnatum* ascocarp (Fig. 2, Fig. S5). The correct hybridization signal of these probes or the general Euk516 probe resulted in clearly colored patches inside the cytoplasm (Fig. 2). Two non-sense probes were used in our study: AS-Euk516 and AS-Rus899, which are inverted and complemented probes of the generalist Euk516 probe or of a *Russula*-specific probe [43], respectively. None of these anti-sense probes showed any hybridization signal. Thus, it did not highlight the hyphal cytoplasm (Fig. 2; Fig. S5), indicating the absence of non-specific hybridization and excluding also a random binding of the fluorochrome. Moreover, in the hybridization experiments conducted without probe addition, no fluorescence was detected at the laser power and detector gain settings used to image the sense probes.

Images collected during the three sets of independent FISH experiments on pure cultures were compared and analyzed (Fig. S5) and images with higher magnification and better resolution are presented with the specific probe T.mag1647 on *T. magnatum* and *T. melanosporum* (Fig. 2).

The hybridization efficiency of each FISH probe is shown in Fig. S5. In particular, the probe T.mag185 (Fig. S5IV) demonstrated a high specificity for *T. magnatum*, and exclusively labeled the target species. This is evidenced by the overlapping signals of the Euk516 probe and the T.mag185 probe when applied to *T. magnatum* gleba tissue (Fig. S5IV-A1) and the absence of random fluorochrome binding with an anti-sense probe (Fig. S5IV-F6). The SSU alignment supported this result, as it revealed at least one single-nucleotide polymorphism (SNP) in all other sequences used for the probe design. However, the probe signal intensity appeared weaker compared to other probes with a similar level of specificity.

In contrast, the probes T.mag645 (Fig. S5III) and T.mag1313 (Fig. S5II) produced more intense signals. Nevertheless, they also recognized other *Tuber* spp. Specifically, the T.mag645 probe detected *T. borchii*, *T. brumale*, and *T. aestivum* (Fig. S5III-B2, C3, D4), as well as species of the *Tuber* and *Choiromyces* (not tested experimentally) genera, displaying an extended hybridization spectrum (Fig. S2). This probe shows no complementarity and hybridization with more distant species (Fig. S2, Fig. S5).

The T.mag1313 probe was much more specific than T.mag645 and showed a positive binding on *T. magnatum* and *T. borchii* only (Fig. S5II-B2). However, a very weak signal was also observed on *T. aestivum* (Fig. S5II-D4). For *T. magnatum*, the signal was also detected but less pronounced than with the other probes, indicating that 18S region has a lower hybridization efficiency. As with the T.mag645 probe, the SSU alignment explained these results since this probe precisely matched the *T. borchii* sequence and exhibited only a single SNP with *T. aestivum*. This likely also explained the very faint signal observed (Fig. S2).

Finally, among the four probes studied, T.mag1647 (Fig. 2, Fig. S5) appears particularly promising for our study, providing a clear hybridization signal, a high degree of specificity for *T. magnatum* without highlighting other *Tuber* spp. or species outside of the *Tuber* genus (Fig. 2, Fig. S5I-A1, B2, C3, D4, E5). During the FISH experiments, this probe showed a better hybridization signal than T.mag185. The T.mag1647 SSU sequence confirmed this specificity, with at least three SNPs differentiating it from other SSU fungal sequences. None of the probes gave positive signal in the hyphae of *M. eximia* or *D. leucomelaena*.

Therefore, we decided to test the T.mag 1647 probe on ectomycorrhizae to further demonstrate its specificity and validate hybridizations on plants. For all the examined ectomycorrhizae of *T. magnatum*, *T. melanosporum*, and *Tuber aestivum*, the binding with the generalist eukaryotic probe Euk516 occurred rarely, indicating that many hyphae of the mantle were inactive. The high specificity of the T.mag1647 probe was also confirmed in *T. magnatum* active hyphae on the ectomycorrhizal mantle (Fig. 2).

### FISH Evidence of Endophytic Colonization

**Fig. 3:**
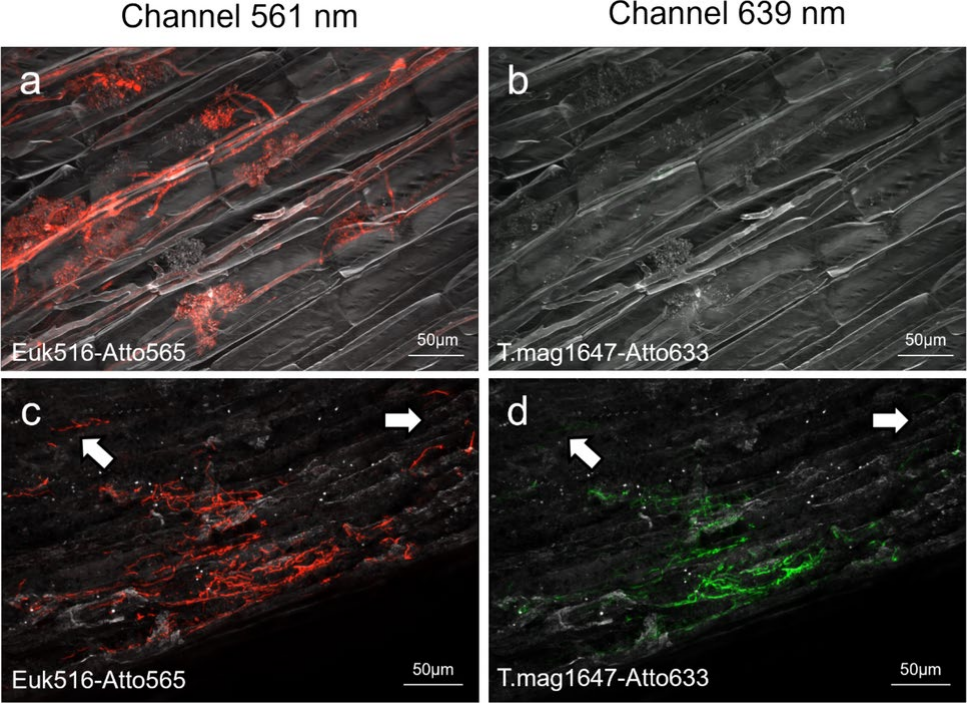
Observation of plant roots by confocal microscopy after Fluorescence In Situ Hybridization (FISH) with the *Tuber magnatum* Picco specific probe T.mag1647 (**b**, **d**; green) and the eukaryotic Euk516 probe (**a**, **c**; red) as a positive control. **a**, **b** Endomycorrhizal fungi found into a *Carex pendula* Huds. root sample with no colocalization of Euk516 and T.mag1647 probes. **c**, **d** Colocalization of Euk516 and T.mag1647 probes into a *C. pendula* roots. The arrows indicate hyphae bonded just with the Euk516 probe. Fluorochrome Atto565 and Atto633 were excited at 561 nm and 639 nm, respectively

After observing all the PCR positive plant samples through FISH and confocal laser scanning microscopy, we successfully observed generic fungal hyphae in all the root samples highlighted by the universal Euk516 probe (Fig. 3a, b). In fact, it was possible to recognize hyphae of endomycorrhizal fungi (Glomeromycota) inside the roots, along with their characteristic arbuscules. However, the latter hyphae were not highlighted by the T.mag1647 *T. magnatum*-specific probes, supporting the specificity of T.mag1647.

In most of the roots examined by FISH with the Euk516 probe, many hyphae were not active (no cytoplasmic labeling). Hyphae specifically marked by the *T. magnatum*-specific probe T.mag1647 were observed in only positive fragments from *C. pendula* (Fig. 3c, d). The root showed in Fig. 3c, d is colonized by two distinct species of endophytic fungus, one of which is *T. magnatum* (perfect colocalization of Euk516 and T.mag1647 probes fluorescence), the other showing only fluorescence from the Euk516 probe (white arrows). Nevertheless, unvital hyphae characterized by suspected *Tuber*-like morphology, as the hyphal thick diameter, septa presence, and absence of clamp-junctions [4, 68], were observed in the inner tissues of PCR-positive plant roots. The overlapping signal between the Euk516 and T.mag1647 probes on the same hyphal region together with their contemporary absence in other sections confirmed the correct binding of the probe. No *T. magnatum* hyphae were observed in fragments negative for *T. magnatum* specific PCR (*n* = 12 observed in total). *Tuber magnatum* hyphae were localized within the roots, probably in the apoplast between cells that were lined by the hyphae and appeared intact.

## Discussion

In this study, specific FISH-probes designed for *T. magnatum* firstly highlighted the presence of this truffle species as an endophyte inside the root of a non-ectomycorrhizal plant raising new questions about its biology. The seasonal sampling of plants conducted in PF has provided key insights into the distribution and dynamics of *T. magnatum* mycelium. Detection of *T. magnatum* by PCR in roots was confined to spring samples from a limited number (21%) of non-ectomycorrhizal plants, mainly herbaceous with the exception of *A. campestre*. No positive PCR was obtained from ectomycorrhizal plant root samples. However, we cannot exclude possible endophytic interactions with ectomycorrhizal plants, considering the limited number of samples examined. The PCR detection of *T. magnatum* only in plant samples collected in spring aligns with previous observations that *T. magnatum* reaches the peak of abundance and the wider distribution of soil mycelium in this season [15]. This further supports that *T. magnatum* mycelium is more active during spring, when the soil water potential is high [71] and allows colonization of the soil and the roots of non-ectomycorrhizal plants. In contrast, other truffle species like *T. aestivum* and *T. melanosporum* were detected inside non-ectomycorrhizal roots in autumn and summer, respectively [25, 26]. This may be related to the different periods of maximum mycelial growth in the soil and, consequently, to ascoma production. Nevertheless, the growth of *T. melanosporum* mycelium can vary significantly depending on the climatic conditions of different years; Queralt et al. [72] observed that the mycelium of *T. melanosporum* began growing in the summer, reached its peak development in the winter, and then declined sharply.

The highly specific FISH-probes designed for *T. magnatum* demonstrated a significant affinity since they successfully labeled the hyphae of *T. magnatum* gleba tissue inside the examined ascoma. Although microscopy and FISH of thick plant tissues are challenging [73], *T. magnatum* specific probes were also able to identify hyphae in the ectomycorrhizal mantle of this species. Interestingly, just a few hyphae appeared viable, similar to findings in *T. aestivum* and *T. melanosporum* ectomycorrhizae, indicating a selective viability among mantle hyphae and/or a possible rapid vitality loss along mantle formation. This characteristic was also observed in other ectomycorrhizal species, where hyphae of the outer layers of the mantle were mostly inactive [42, 74]. Similarly, the endophytic hyphae of *T. magnatum* colonizing roots during spring might rapidly lose their vitality inside the plant tissue. Alternatively, endophytic hyphae have low metabolic activity inside host tissues and reach undetectable levels, which could explain both the restricted season during which they can be found inside the roots and the discrepancy between the number of PCR-positive results and FISH observations. In fact, FISH applied to herbaceous plants yielded only one *T. magnatum* positive detection on *C. pendula* roots, whereas 21 positive plant samples were found using species-specific PCRs. Of course, difficulty of visualization (including the fact that PCR with *T. magnatum* specific primers were carried out on a different, although close, piece of root) can also contribute to this discrepancy. Moreover, contamination of the rhizoplane by *T. magnatum* mycelium cannot be entirely ruled out; however, the external mycelium of *T. magnatum* has never been observed on either PCR-positive or PCR-negative root fragments when using the FISH technique. This further supports evidence of a loss of hyphal vitality within the PCR-positive roots although it also warrants further exploration.

It is notable that the only positive plant species, *C. pendula*, is one of the most prevalent herbaceous plants in the PF [75]. Other species of the same genus, *Carex muricata* L. and *Carex flacca* Schreb., were found to be associated with *T. aestivum* and *T. melanosporum*, respectively [25, 76]. Based on molecular methods, these truffles share some endophytic partners of the same species or genus with *T. magnatum*. For instance, *H. helix* appears capable of hosting *T. aestivum*, *T. melanosporum*, and *T. magnatum* [25, 26]. Similarly, the genus *Viola* is shared among these three truffle species. However, most of the *T. magnatum* endophytic host plant species found in this study are not shared with the other two truffles in the current (limited) knowledge [25, 26, 76]. Only *T. aestivum* and *T. magnatum* share the genus *Taraxacum* as a non-ectomycorrhizal host.

Without excluding the possible influence of annual weather variations and specific fungal ecological characteristics, *T. magnatum* appears to be more restrictive as an endophyte within non-ectomycorrhizal host plant roots, interacting with a reduced number of individuals and plant species (21% of the collected plant samples and 31% of the examined species). Other *Tuber* species, such as *T. melanosporum* and *T. aestivum*, were more frequent and widespread within non-ectomycorrhizal plant species [25, 26, 76]. For instance, *T. aestivum* was positively detected in 54% of collected plant samples and 80% of examined plant species [26]. Regarding *T. melanosporum*, Schneider-Maunoury et al. [25] reported a very high percentage of non-ectomycorrhizal plant colonization, with 79% of collected plant samples and 90% of plant species. In a subsequent study, *T. melanosporum* was detected in 53% of the collected plant samples and 90% of the examined plant species by PCR, and its presence was subsequently confirmed by FISH in the four most colonized species [26]. The endophytic ability of *T. magnatum* further supports the idea that such a trait is widespread among *Tuber* spp., as well as other ectomycorrhizal fungi [21–27, 43, 77], and is also congruent to the hypothesis that endophytism may have predated the emergence of ectomycorrhizal abilities in fungi (the Waiting Room Hypothesis, where endophytism is a waiting room to closer symbiosis [26, 78]).

A possible explanation for the reduced endophytic abundance of *T. magnatum* compared to other truffle species may lie in the differences in mycelial biomass and in the vertical soil distribution of its mycelium. The mycelial biomass density of *T. aestivum* and *T. melanosporum* in soil appears to be up to three orders of magnitude higher than that of *T. magnatum* [15, 24, 72]. In particular, *T. aestivum* seems to produce relatively dense soil mycelium compared to other ectomycorrhizal fungi [15, 24]. Furthermore, the density of *T. aestivum* mycelium on the root surface of ectomycorrhizal plants appears to be very high and may be comparable to that found in radical portions provided with ectomycorrhizae. This type of association between *T. aestivum* mycelium and root surfaces other than ectomycorrhizae could explain its tendency to also interact with the roots of non-ectomycorrhizal plant species.

Regarding the vertical distribution of the mycelium, as suggested by Ceruti [79] and Iotti et al. [71], the mycelium of *T. magnatum* may extend deeper into the soil than that of other ectomycorrhizal species [80–83], particularly under stress conditions such as very dry winters or summers [84]. During the sampling years, especially in 2022, winter seasons were exceptionally dry [85, 86], which may have caused the mycelium to develop at greater depths. The roots of non-ectomycorrhizal plant species usually grow in the upper soil layer, and *T. magnatum* mycelium might encounter difficulties in reaching them through dry soil layers.

In summary, our results seem to support the idea that *T. magnatum* mycelium exists within non-ectomycorrhizal plant roots, but it may be rare. Mycelial biomass within herbaceous roots is likely insufficient to play a major role in supporting the nutritional requirements of *T. magnatum* to colonize the soil and even less to fruit. While symbiotic *Bradyrhizobia* may contribute nitrogen to the *T. magnatum* mycelium and ascoma [16, 87], the nutritional role of non-ectomycorrhizal wild plants in carbon uptake remains unclear and requires further investigation. Nevertheless, the endophytic colonization of non-ectomycorrhizal plants by *T. magnatum* may be involved in other ecological mechanisms, such as protection of hyphae or habitat exploration. On the latter issue, Zampieri et al. [14] molecularly identified the *T. magnatum* mycelium in soil up to 100 m from the nearest productive tree. This considerable distance could have been traversed over a long period, with non-ectomycorrhizal plants serving as temporary refuges during the mycelial migration.

The highly specific FISH probes designed in this study open up new possibilities for further investigating the relationships between *T. magnatum* mycelium and plants, soil, and other organisms, emphasizing the role of vital hyphae while excluding those detected by relic *T. magnatum* DNA. Further investigations will be necessary to confirm the endophytic tendencies of *T. magnatum* and to understand the potential role this association plays in its life cycle and biology. It remains unclear whether the endophytism of *T. magnatum* is beneficial for the fungus, the host plant, or both. Assuming a beneficial interaction from endophytic colonization by *T. magnatum* in both ectomycorrhizal and non-ectomycorrhizal host plant species, this could support the introduction of these plants to enhance white truffle plantations.

## Supplementary Information

Below is the link to the electronic supplementary material.

Supplementary file1 (DOCX 14478 KB)

## Data Availability

All data generated or analyzed during this study are included in this published article and its supplementary material files. ITS partial sequences of *T*. *magnatum* ectomycorrhizae were deposited at NCBI (GenBank) with the following accession numbers: PQ818236 to PQ818245.
