# Supplementary material for: Newly Designed Fluorescence In Situ Hybridization Probes Reveal Previously Unknown Endophytic Abilities of Tuber magnatum in Herbaceous Plants
Source: Microb Ecol. 2025 May 8;88:42. doi: 10.1007/s00248-025-02542-z (PMC12062114; doi:10.1007/s00248-025-02542-z)
Supplement: Supplementary file 1 [file 248_2025_2542_MOESM1_ESM.docx]

**Newly designed Fluorescence *In situ* Hybridization probes reveal previously unknown endophytic abilities of *Tuber magnatum* in herbaceous plants**

**Microbial Ecology**

Simone Graziosi^1*(^ ^https://orcid.org/0000-0003-3117-6474)^, Lara Deloche^2(https://orcid.org/0009-0003-4783-7844)^, Mélanie Januario^2^, Marc-André Selosse^2,3,4(https://orcid.org/0000-0003-3471-9067)^, Aurélie Deveau^5(https://orcid.org/0000-0001-6266-5241)^, Cyrille Bach^5(https://orcid.org/0000-0002-3987-3226)^, Zhixiao Chen^5(https://orcid.org/0009-0005-6257-6851)^, Claude Murat^5(https://orcid.org/0000-0003-0685-7307)^, Mirco Iotti^6(https://orcid.org/0000-0003-2705-0385)^, Philippe Rech^2**(https://orcid.org/0000-0002-6631-9888)^, Alessandra Zambonelli^1(https://orcid.org/0000-0003-1710-7069)^

^1^Department of Agricultural and Food Sciences, University of Bologna, Viale G. Fanin 44, 40127, Bologna, Italy

^2^ ISYEB, Muséum national d’Histoire naturelle, CNRS, EPHE-PSL, Sorbonne Université, 57 rue Cuvier, CP39, 75005 Paris, France

^3^Department of Plant Taxonomy and Nature Conservation, University of Gdańsk, ul. Wita Stwosza 59, 80-308 Gdańsk, Poland

^4^Institut Universitaire de France, Paris, France

^5^Université de Lorraine, INRAE, IAM, F-54000 Nancy, France

^6^Department of Life, Health and Environmental Science, University of L'Aquila, Via Vetoio, 67100, Coppito, L'Aquila, Italy

***Corresponding author**: simone.graziosi5@unibo.it

****Co-corresponding author:** philippe.rech@mnhn.fr

**Table S 1** Description of sampling areas and sampling dates*.*

| **Sampling Area** | **Altitude a.s.l (m)** | **Average annual temperature (°C)^a^** | **Average annual precipitation (mm)^b^** | **Soil**  **Classification^c^** | **Putative**  **host plants** |
| --- | --- | --- | --- | --- | --- |
| Città della Pieve – CDP (PG) | 455 - 430 | 15.6 | 825.3 | *Typic Haplustepts fine-silty, mixed, mesic* | *Corylus avellana L., Ostrya carpinifolia Scop., Populus nigra L., Populus alba L., Quercus cerris L., Quercus pubescens Willd., Quercus robur L., Salix spp.* |
| Panfilia Forest – PF (FE) | 30 | 13.85 | 889 | *Oxyaquic Ustifluvents, fine-silty, mixed, active, calcareous, mesic* | *C. avellana*, *P. alba*, *Q. robur*, *Salix* spp., *Tilia cordata* Mill. |
| Montefalcone nel Sannio – MNS (CB) | 345 | 13.5 | 782.3 | *Vertic Calciustept* | *C. avellana* L., *O. carpinifolia* Scop., *P. alba* L., *Q. cerris* L., *Q. pubescens* Willd., *Q. robur* L. |

Note**:** ^a,b^ The climatic data were provided by https://www.regione.umbria.it for Città della Pieve (CDP) (2006 – 2015), https://www.arpae.it for Panfilia Forest (PF) (1991 – 2020), https://www.istat.it for Montefalcone nel Sannio (MNS) (2006 – 2022) ^c^USDA soil taxonomy, the soil data were provided by https://www.umbriageo.regione.umbria.it for Città della Pieve (CDP), https://www.ambiente.regione.emilia-romagna.it/it for Panfilia Forest (PF), https://www.arsarp.it for Montefalcone nel Sannio (MNS).

**Table S 2** Oligonucleotide primers and PCR cycling conditions used in this study.

| Primer | Sequence (5’ – 3’) | Target gene | PCR cycling conditions | Reference |
| --- | --- | --- | --- | --- |
| ITS1f | TCCGTAGGTGAACCTGCGG | ITS rRNA | 95 °C 6 min; 34 x (94 °C 30 s, 56 °C 30 s, 72 °C 1 min), 72 °C 7 min | White et al., 1990; Gardes and Bruns 1993 |
| ITS4 | TCCTCCGCTTATTGATATGC | ITS rRNA |  |  |
| ITS1f | TCCGTAGGTGAACCTGCGG | ITS rRNA | 95 °C 6 min; 35 x (95 °C 30 s, 60 °C 40 s, 72 °C 40 s), 72 °C 5 min |  |
| ITS2 | GCTGCGTTCTTCATCGATGC | ITS rRNA |  |  |
| TmagI | GGATGCGTCTCCGAATCCTGAAT | ITS rRNA | 94 °C 5 min; 25 x (94 °C 20 s, 62 °C 15 s, 72 °C 30 sec), 72 °C 7 min | Amicucci et al., 1998 |
| TmagII | TCGGGCCCTTTCTCAGACTGCTG | ITS rRNA |  |  |

**Table S 3** 18S sequences employed for *Tuber magnatum* Picco FISH specific probes design. Sequences were obtained from genomic data published in NCBI GenBank (NCBI, https://www.ncbi.nlm.nih.gov).

| **Species** | **Order** | **Family** | **Accession number NCBI** | **Geographical origin** | **References** |
| --- | --- | --- | --- | --- | --- |
| *Balsamia magnata* Harkn. | Pezizales | Helvellaceae | U42656.1 | USA, California | O'Donnell et al., 1997 |
| *Balsamia vulgaris* Vittad. | Pezizales | Helvellaceae | AF054905.1 | Italy, Emilia-Romagna, Bologna | Percudani et al., 1999 |
| *Choiromyces meandriformis* Vittad. | Pezizales | Tuberaceae | AF054904.1 | Italy, Emilia-Romagna, Bologna | Percudani et al., 1999 |
| *Choiromyces venosus* (Fr.) Th. Fr. | Pezizales | Tuberaceae | U42661.1 | USA, Oregon | O'Donnell et al., 1997 |
| *Cryomyces antarcticus* Selbmann, de Hoog, Mazzaglia, Friedmann & Onofri | Dothideomycetes | *incertae sedis* | NG_061042.1 | Linnaeus Terrace, McMurdo Dry Valleys, Southern Victoria Land, Antarctica | Selbmann et al., 2005 |
| *Cryomyces montanus* Isola & Zucconi | Dothideomycetes *incertae sedis* | NA | NG_070634.1 | Unknown | Ruibal et al., 2009; Selbmann et al., 2005 |
| *Dingleya verrucosa* Trappe | Pezizales | Tuberaceae | U42659.1 | New Zealand | O'Donnell et al., 1997 |
| *Dothidea sambuci* Dothidea sambuci (Pers.) Fr. | Dothideales | Dothideaceae | NG_012432.1 | Austria, Stübinggraben | Unpublished |
| *Dothiora cannabinae* Froid. | Dothideales | Dothioraceae | NG_062696.1 | Unknown | Spatafora et al., 2006 |
| *Eleutherascus lectardii* (Nicot) Arx | Pezizales | Ascodesmidaceae | NG_062685.1 | France, Moselle | Hansen et al., 2005 |
| *Helvella compressa* (Snyder) N.S. Weber | Pezizales | Helvellaceae | AY544699.1 | Unknown | Unpublished |
| *Helvella lacunosa* Afzel. | Pezizales | Helvellaceae | U42654.1 | USA, Oregon | O'Donnell et al., 1997 |
| *Karstenella vernalis* Harmaja | Pezizales | Karstenellaceae | FJ499392.1 | Finland | Hansen et al., 2008 |
| *Labyrinthomyces varius* (Rodway) Trappe | Pezizales | Tuberaceae | U42662.1 | Australia | O'Donnell et al., 1997 |
| *Lecophagus longisporus* (G.L. Barron, C. Morik. & Saikawa) Y. Tanabe, Nagah., Saikawa & Sugiy. | Orbiliales | Orbiliales *incertae sedis* | NG_062600.1 | Unknown | Tanabe et al., 1999 |
| *Meristemomyces frigidus* Isola & Onofri | Mycosphaerellales | Teratosphaeriaceae | NG_061096.1 | Unknown | Ruibal et al., 2009 |
| *Morchella elata* Fr. | Pezizales | Morchellaceae | U42641.1 | USA, Michigan | O'Donnell et al., 1997 |
| *Morchella esculenta* (L.) Pers. | Pezizales | Morchellaceae | U42642.1 | USA, Oregon | O'Donnell et al., 1997 |
| *Pseudogymnoascus destructans* (Blehert & Gargas) Minnis & D.L. Lindner | Thelebolales | Thelebolaceae | NG_065563.1 | USA, New York | Blehert et al., 2009; Gargas et al., 2009 |
| *Ramoconidiophora euphorbiae* (S. Nasr, S. Bien & Damm) S. Bien & Damm | Phacidiales | Phacidiales *incertae sedis* | NG_067663.1 | Iran | Nasr et al., 2018 |
| *Reddellomyces donkii* (Malençon) Trappe, Castellano & Malajczuk | Pezizales | Tuberaceae | U42660.1 | USA, California | O'Donnell et al., 1997 |
| *Scytalidium lignicola* Pesante | Leotiomycetes *incertae sedis* | NA | NG_061078.1 | Italy | Kang et al., 2010 |
| *Symbiotaphrina buchneri* Gräbner ex W. Gams & Arx | Symbiotaphrinales | Symbiotaphrinaceae | NG_081375.1 | Unknown | Suh et al., 2003;  Suh et al., 2005 |
| *Symbiotaphrina kochii* Jurzitza ex W. Gams & Arx | Symbiotaphrinales | Symbiotaphrinaceae | NG_062647.1 | Unknown | Suh et al., 2005 |
| *Tuber aestivum* Vittad. | Pezizales | Tuberaceae | AM748740.1 | Italy | Jeandroz et al., 2008 |
| *Tuber borchii* Vittad. | Pezizales | Tuberaceae | AF054902.1 | Italy, Piedmont, Turin | Percudani et al., 1999 |
| *Tuber brumale* Vittad. | Pezizales | Tuberaceae | AM748734.1 | Italy | Jeandroz et al., 2008 |
| *Tuber excavatum* Vittad. | Pezizales | Tuberaceae | X98089.1 | Unknown | Unpublished |
| *Tuber gibbosum* Harkn. | Pezizales | Tuberaceae | U42663.1 | USA, Oregon | O'Donnell et al., 1997 |
| *Tuber indicum* Cooke & Massee | Pezizales | Tuberaceae | AM748737.1 | China, Yunnan | Jeandroz et al., 2008 |
| *Tuber magnatum* Picco | Pezizales | Tuberaceae | AF054901.1 | Italy, Emilia-Romagna, Bologna | Percudani et al., 1999 |
| *Tuber melanosporum* Vittad. | Pezizales | Tuberaceae | AM748736.1 | Italy, Piedmont | Jeandroz et al., 2008 |
| *Tuber oligospermum* (Tul. & C. Tul.) Trappe | Pezizales | Tuberaceae | AM748738.1 | Marocco | Jeandroz et al., 2008 |
| *Tuber panniferum* Tul. & C. Tul. | Pezizales | Tuberaceae | AF054903.1 | Italy, Tuscany, Siena | Percudani et al., 1999 |
| *Tuber pseudoexcavatum* Y. Wang, G. Moreno, Riousset, Manjón & G. Riousset | Pezizales | Tuberaceae | AM748735.1 | China, Sichuan | Jeandroz et al., 2008 |
| *Tuber puberulum* Berk. & Broome | Pezizales | Tuberaceae | AM748739.1 | Unknown | Jeandroz et al., 2008 |
| *Tuber rapaeodorum* Tul. & C. Tul. | Pezizales | Tuberaceae | Z49755.1 | Unknown | Landvik and Eriksson 1994 |
| *Uncultured ectomycorrhiza (Tuberaceae)* | Pezizales | Tuberaceae | AY940164.1 | Italy, Umbria | Baciarelli-Falini et al., 2006 |
| *Uncultured mycorrhiza (Tuberaceae)* | Pezizales | Tuberaceae | DQ402510.1 | Italy, Umbria | Baciarelli-Falini et al., 2006 |
| *Underwoodia columnaris* Peck | Pezizales | Helvellaceae | U42658.1 | USA, Michigan | O'Donnell et al., 1997 |

Note: NA = not available.

**Table S 4** 18S rRNA sequence probes tested in this study.

| Probe name^a^ | Sequence | Fluorescent dye | NI(%)^b^ |
| --- | --- | --- | --- |
| Euk516 | ACCAGACTTGCCCTCC | Atto565 | - |
| AS-Euk516 ^a^ | GGAGGGCAAGTCTGGT | Atto633 | - |
| T.mag185 | CCAAGGAGGCCGAGGCCGTT | Atto633 | 75% |
| T.mag645 | GCGGCTAGCCAGAAGGAAAGATCCG | Atto633 | 68% |
| T.mag1313 | CAGCGGCCCGCGAGATGCGGGTCGG | Atto633 | 88% |
| T.mag1647 | GGCCGTTGCCAACCTCCGCAGGGC | Atto633 | 75% |
| AS-Rus899 ^a^ | ATTTACGCAAGACAAACTATTGCG | Atto633 | - |

Note: ^a^ = AS: anti-sense probe used as a negative “anti-sense” control, ^b^ = Maximum percentage of nucleotide identity with other 18S rRNA sequences of *Tuber spp.* tested (except *Tuber magnatum* Picco)

**Table S 5.** List of plant species collected in the fruiting sites. The presence of *Tuber magnatum* Picco in the soil was molecularly verified in all sites.

| **Nr.** | **Species^a^** | **Season** | **Fruiting site** | **Point** | **Sampling data** |
| --- | --- | --- | --- | --- | --- |
| **1** | *Arum italicum* Mill. | Spring | Città della Pieve (PG) | 1 | 17/03/2023 |
| **2** | ***Arum italicum* Mill.** | Spring | Città della Pieve (PG) | 1 | 17/03/2023 |
| **3** | ***Urtica dioica* L.** | Spring | Città della Pieve (PG) | 1 | 17/03/2023 |
| **4** | ***Hedera helix* L.** | Spring | Città della Pieve (PG) | 1 | 17/03/2023 |
| **5** | *Brassica juncea* (L.) Czern. | Spring | Città della Pieve (PG) | 1 | 17/03/2023 |
| **6** | ***Urtica dioica* L.** | Spring | Città della Pieve (PG) | 1 | 17/03/2023 |
| **7** | *Crepis* sp. | Spring | Città della Pieve (PG) | 1 | 17/03/2023 |
| **8** | ***Arum italicum* Mill.** | Spring | Città della Pieve (PG) | 1 | 17/03/2023 |
| **9** | *Urtica dioica L.* | Spring | Città della Pieve (PG) | 1 | 17/03/2023 |
| **10** | *Sison amomum* L. | Spring | Città della Pieve (PG) | 1 | 17/03/2023 |
| **1** | ***Primula vulgaris* Huds.** | Spring | Città della Pieve (PG) | 2 | 17/03/2023 |
| **2** | ***Viola odorata* L.** | Spring | Città della Pieve (PG) | 2 | 17/03/2023 |
| **3** | ***Primula vulgaris* Huds.** | Spring | Città della Pieve (PG) | 2 | 17/03/2023 |
| **4** | ***Ranunculus bulbosus* L.** | Spring | Città della Pieve (PG) | 2 | 17/03/2023 |
| **5** | ***Hedera helix*** L. | Spring | Città della Pieve (PG) | 2 | 17/03/2023 |
| **6** | ***Primula vulgaris* Huds*.*** | Spring | Città della Pieve (PG) | 2 | 17/03/2023 |
| **7** | ***Hedera helix* L.** | Spring | Città della Pieve (PG) | 2 | 17/03/2023 |
| **8** | *Ranunculus bulbosus* L. | Spring | Città della Pieve (PG) | 2 | 17/03/2023 |
| **9** | *Arum italicum* Mill. | Spring | Città della Pieve (PG) | 2 | 17/03/2023 |
| **10** | ***Galium verrucosum* Huds.** | Spring | Città della Pieve (PG) | 2 | 17/03/2023 |
| **1** | *Tussilago farfara* L. | Spring | Città della Pieve (PG) | 3 | 17/03/2023 |
| **2** | ***Taraxacum dissectum* (Ledeb.) Ledeb.** | Spring | Città della Pieve (PG) | 3 | 17/03/2023 |
| **3** | *Taraxacum dissectum* (Ledeb.) Ledeb. | Spring | Città della Pieve (PG) | 3 | 17/03/2023 |
| **4** | ***Sison amomum* L.** | Spring | Città della Pieve (PG) | 3 | 17/03/2023 |
| **5** | ***Hedera helix* L.** | Spring | Città della Pieve (PG) | 3 | 17/03/2023 |
| **6** | *Verbascum phlomoides* L. | Spring | Città della Pieve (PG) | 3 | 17/03/2023 |
| **7** | *Picris hieracioides* L. | Spring | Città della Pieve (PG) | 3 | 17/03/2023 |
| **8** | ***Hedera helix*** L. | Spring | Città della Pieve (PG) | 3 | 17/03/2023 |
| **9** | *Primula vulgaris* Huds. | Spring | Città della Pieve (PG) | 3 | 17/03/2023 |
| **10** | ***Hedera helix*** L*.* | Spring | Città della Pieve (PG) | 3 | 17/03/2023 |
| **1** | *Primula vulgaris* Huds. | Spring | Città della Pieve (PG) | 4 | 17/03/2023 |
| **2** | *Centaurea benedicta* (L.) L. | Spring | Città della Pieve (PG) | 4 | 17/03/2023 |
| **3** | *Phragmites australis* (Cav.) Trin. ex Steud. | Spring | Città della Pieve (PG) | 4 | 17/03/2023 |
| **4** | *Verbascum phlomoides* L. | Spring | Città della Pieve (PG) | 4 | 17/03/2023 |
| **5** | *Geranium purpureum* Vill. | Spring | Città della Pieve (PG) | 4 | 17/03/2023 |
| **6** | *Helminthotheca echioides* (L.) Holub | Spring | Città della Pieve (PG) | 4 | 17/03/2023 |
| **7** | *Ranunculus bulbosus* L. | Spring | Città della Pieve (PG) | 4 | 17/03/2023 |
| **8** | *Ranunculus bulbosus* L. | Spring | Città della Pieve (PG) | 4 | 17/03/2023 |
| **9** | *Geranium purpureum* Vill. | Spring | Città della Pieve (PG) | 4 | 17/03/2023 |
| **10** | *Viola odorata* L. | Spring | Città della Pieve (PG) | 4 | 17/03/2023 |
| **1** | *Arum italicum* Mill. | Spring | Città della Pieve (PG) | 5 | 17/03/2023 |
| **2** | *Poterium sanguisorba* L. | Spring | Città della Pieve (PG) | 5 | 17/03/2023 |
| **3** | *Primula vulgaris* Huds. | Spring | Città della Pieve (PG) | 5 | 17/03/2023 |
| **4** | *Poterium sanguisorba* L. | Spring | Città della Pieve (PG) | 5 | 17/03/2023 |
| **5** | ***Hedera helix*** L. | Spring | Città della Pieve (PG) | 5 | 17/03/2023 |
| **6** | *Viola odorata* L. | Spring | Città della Pieve (PG) | 5 | 17/03/2023 |
| **7** | *Primula vulgaris Huds*. | Spring | Città della Pieve (PG) | 5 | 17/03/2023 |
| **8** | *Arum italicum* Mill. | Spring | Città della Pieve (PG) | 5 | 17/03/2023 |
| **9** | *Ageratum houstonianum* Mill. | Spring | Città della Pieve (PG) | 5 | 17/03/2023 |
| **10** | *Ageratum houstonianum* Mill. | Spring | Città della Pieve (PG) | 5 | 17/03/2023 |
| **1** | *Primula vulgaris Huds.* | Spring | Città della Pieve (PG) | 6 | 17/03/2023 |
| **2** | *Primula vulgaris Huds.* | Spring | Città della Pieve (PG) | 6 | 17/03/2023 |
| **3** | *Geranium purpureum* Vill. | Spring | Città della Pieve (PG) | 6 | 17/03/2023 |
| **4** | *Erodium malacoides* (L.) L'Hér. | Spring | Città della Pieve (PG) | 6 | 17/03/2023 |
| **5** | *Hypericum perforatum* L. | Spring | Città della Pieve (PG) | 6 | 17/03/2023 |
| **6** | *Erodium malacoides* (L.) L'Hér. | Spring | Città della Pieve (PG) | 6 | 17/03/2023 |
| **7** | *Helminthotheca echioides* (L.) Holub | Spring | Città della Pieve (PG) | 6 | 17/03/2023 |
| **8** | *Helminthotheca echioides* (L.) Holub | Spring | Città della Pieve (PG) | 6 | 17/03/2023 |
| **9** | *Cerastium fontanum* Baumg. | Spring | Città della Pieve (PG) | 6 | 17/03/2023 |
| **10** | *Verbascum phlomoides* L. | Spring | Città della Pieve (PG) | 6 | 17/03/2023 |
| **1** | *Viola odorata* L. | Spring | Montefalcone n.S. (CB) | 1 | 26/04/2023 |
| **2** | *Ranunculus* sp. | Spring | Montefalcone n.S. (CB) | 1 | 26/04/2023 |
| **3** | *Centaruea benedicta* (L.) L. | Spring | Montefalcone n.S. (CB) | 1 | 26/04/2023 |
| **4** | ***Aiuga reptans* L.** | Spring | Montefalcone n.S. (CB) | 1 | 26/04/2023 |
| **5** | *Aiuga reptans* L. | Spring | Montefalcone n.S. (CB) | 1 | 26/04/2023 |
| **6** | *Muscari comosum* (L.) Mill. | Spring | Montefalcone n.S. (CB) | 1 | 26/04/2023 |
| **7** | *Pimpinella saxifraga* L. | Spring | Montefalcone n.S. (CB) | 1 | 26/04/2023 |
| **8** | *Ranunculus* sp. | Spring | Montefalcone n.S. (CB) | 1 | 26/04/2023 |
| **9** | *Ranunculus* sp. | Spring | Montefalcone n.S. (CB) | 1 | 26/04/2023 |
| **10** | *Ranunculus* sp. | Spring | Montefalcone n.S. (CB) | 1 | 26/04/2023 |
| **1** | *Corylus avellana* L. | Winter | Panfilia Forest (FE) | 1 | 23/02/2022 |
| **2** | ***Hedera helix*** L. | Winter | Panfilia Forest (FE) | 1 | 23/02/2022 |
| **3** | *Rubus caesus* L. | Winter | Panfilia Forest (FE) | 1 | 23/02/2022 |
| **4** | *Rubus caesus* L. | Winter | Panfilia Forest (FE) | 1 | 23/02/2022 |
| **5** | ***Hedera helix*** L. | Winter | Panfilia Forest (FE) | 1 | 23/02/2022 |
| **6** | *Rubus caesus* L. | Winter | Panfilia Forest (FE) | 1 | 23/02/2022 |
| **7** | *Carex pendula* Huds*.* | Winter | Panfilia Forest (FE) | 1 | 23/02/2022 |
| **8** | *Carex pendula* Huds*.* | Winter | Panfilia Forest (FE) | 1 | 23/02/2022 |
| **9** | *Rubus caesus* L. | Winter | Panfilia Forest (FE) | 1 | 23/02/2022 |
| **10** | ***Hedera helix*** L. | Winter | Panfilia Forest (FE) | 1 | 23/02/2022 |
| **1** | *Carex pendula* Huds | Winter | Panfilia Forest (FE) | 2 | 23/02/2022 |
| **2** | *Rubus caesus* L. | Winter | Panfilia Forest (FE) | 2 | 23/02/2022 |
| **3** | *Carex pendula* Huds | Winter | Panfilia Forest (FE) | 2 | 23/02/2022 |
| **4** | *Rubus caesus* L. | Winter | Panfilia Forest (FE) | 2 | 23/02/2022 |
| **5** | *Carex pendula* Huds | Winter | Panfilia Forest (FE) | 2 | 23/02/2022 |
| **6** | *Populus alba* L. | Winter | Panfilia Forest (FE) | 2 | 23/02/2022 |
| **7** | *Rubus caesus* L. | Winter | Panfilia Forest (FE) | 2 | 23/02/2022 |
| **8** | *Carex pendula* Huds | Winter | Panfilia Forest (FE) | 2 | 23/02/2022 |
| **9** | *Carex pendula* Huds | Winter | Panfilia Forest (FE) | 2 | 23/02/2022 |
| **10** | *Rubus caesus* L. | Winter | Panfilia Forest (FE) | 2 | 23/02/2022 |
| **1** | *Carex pendula* Huds | Winter | Panfilia Forest (FE) | 3 | 23/02/2022 |
| **2** | ***Hedera helix*** L. | Winter | Panfilia Forest (FE) | 3 | 23/02/2022 |
| **3** | *Carex pendula* Huds | Winter | Panfilia Forest (FE) | 3 | 23/02/2022 |
| **4** | *Sambucus nigra* L. | Winter | Panfilia Forest (FE) | 3 | 23/02/2022 |
| **5** | *Rubus caesus* L. | Winter | Panfilia Forest (FE) | 3 | 23/02/2022 |
| **6** | ***Hedera helix*** L. | Winter | Panfilia Forest (FE) | 3 | 23/02/2022 |
| **7** | *Cornus mas* L. | Winter | Panfilia Forest (FE) | 3 | 23/02/2022 |
| **8** | *Carex pendula* Huds | Winter | Panfilia Forest (FE) | 3 | 23/02/2022 |
| **9** | *Acer campestre* L. | Winter | Panfilia Forest (FE) | 3 | 23/02/2022 |
| **10** | ***Hedera helix*** L. | Winter | Panfilia Forest (FE) | 3 | 23/02/2022 |
| **1** | ***Hedera helix*** L. | Spring | Panfilia Forest (FE) | 1 | 12/04/2022 |
| **2** | ***Hedera helix*** L. | Spring | Panfilia Forest (FE) | 1 | 12/04/2022 |
| **3** | ***Hedera helix*** L. | Spring | Panfilia Forest (FE) | 1 | 12/04/2022 |
| **4** | ***Hedera helix*** L*.* | Spring | Panfilia Forest (FE) | 1 | 12/04/2022 |
| **5** | ***Carex pendula* Huds*.*** | Spring | Panfilia Forest (FE) | 1 | 12/04/2022 |
| **6** | ***Carex pendula* Huds*.*** | Spring | Panfilia Forest (FE) | 1 | 12/04/2022 |
| **7** | ***Carex pendula* Huds.** | Spring | Panfilia Forest (FE) | 1 | 12/04/2022 |
| **8** | *Rubus caesus* L. | Spring | Panfilia Forest (FE) | 1 | 12/04/2022 |
| **9** | ***Hedera helix*** L. | Spring | Panfilia Forest (FE) | 1 | 12/04/2022 |
| **10** | *Corylus avellana* L. | Spring | Panfilia Forest (FE) | 1 | 12/04/2022 |
| **1** | *Populus alba* L. | Spring | Panfilia Forest (FE) | 2 | 12/04/2022 |
| **2** | *Rubus caesus* L. | Spring | Panfilia Forest (FE) | 2 | 12/04/2022 |
| **3** | *Carex pendula* Huds*.* | Spring | Panfilia Forest (FE) | 2 | 12/04/2022 |
| **4** | *Carex pendula* Huds*.* | Spring | Panfilia Forest (FE) | 2 | 12/04/2022 |
| **5** | ***Carex pendula* Huds***.* | Spring | Panfilia Forest (FE) | 2 | 12/04/2022 |
| **6** | *Carex pendula* Huds*.* | Spring | Panfilia Forest (FE) | 2 | 12/04/2022 |
| **7** | *Rubus caesus* L. | Spring | Panfilia Forest (FE) | 2 | 12/04/2022 |
| **8** | *Carex pendula* Huds*.* | Spring | Panfilia Forest (FE) | 2 | 12/04/2022 |
| **9** | *Carex pendula* Huds*.* | Spring | Panfilia Forest (FE) | 2 | 12/04/2022 |
| **10** | *Viburnum lantana* L. | Spring | Panfilia Forest (FE) | 2 | 12/04/2022 |
| **1** | ***Acer campestre* L.** | Spring | Panfilia Forest (FE) | 3 | 12/04/2022 |
| **2** | *Cornus mas* L. | Spring | Panfilia Forest (FE) | 3 | 12/04/2022 |
| **3** | ***Hedera helix*** L. | Spring | Panfilia Forest (FE) | 3 | 12/04/2022 |
| **4** | *Carex pendula* Huds. | Spring | Panfilia Forest (FE) | 3 | 12/04/2022 |
| **5** | *Carex pendula* Huds. | Spring | Panfilia Forest (FE) | 3 | 12/04/2022 |
| **6** | *Sambucus nigra* L. | Spring | Panfilia Forest (FE) | 3 | 12/04/2022 |
| **7** | ***Hedera helix*** L. | Spring | Panfilia Forest (FE) | 3 | 12/04/2022 |
| **8** | *Carex pendula* Huds. | Spring | Panfilia Forest (FE) | 3 | 12/04/2022 |
| **9** | *Tilia cordata* Mill. | Spring | Panfilia Forest (FE) | 3 | 12/04/2022 |
| **10** | ***Hedera helix*** L*.* | Spring | Panfilia Forest (FE) | 3 | 12/04/2022 |
| **1** | *Rubus caesus* L. | Summer | Panfilia Forest (FE) | 1 | 23/07/2022 |
| **2** | *Corylus avellana* L. | Summer | Panfilia Forest (FE) | 1 | 23/07/2022 |
| **3** | ***Hedera helix*** L. | Summer | Panfilia Forest (FE) | 1 | 23/07/2022 |
| **4** | ***Hedera helix*** L. | Summer | Panfilia Forest (FE) | 1 | 23/07/2022 |
| **5** | *Carex pendula* Huds*.* | Summer | Panfilia Forest (FE) | 1 | 23/07/2022 |
| **6** | ***Hedera helix*** L. | Summer | Panfilia Forest (FE) | 1 | 23/07/2022 |
| **7** | *Carex pendula* Huds*.* | Summer | Panfilia Forest (FE) | 1 | 23/07/2022 |
| **8** | *Carex pendula* Huds*.* | Summer | Panfilia Forest (FE) | 1 | 23/07/2022 |
| **9** | ***Hedera helix*** L. | Summer | Panfilia Forest (FE) | 1 | 23/07/2022 |
| **10** | ***Hedera helix*** L. | Summer | Panfilia Forest (FE) | 1 | 23/07/2022 |
| **1** | *Rubus caesus* L. | Summer | Panfilia Forest (FE) | 2 | 23/07/2022 |
| **2** | ***Hedera helix*** L. | Summer | Panfilia Forest (FE) | 2 | 23/07/2022 |
| **3** | ***Hedera helix*** L. | Summer | Panfilia Forest (FE) | 2 | 23/07/2022 |
| **4** | ***Hedera helix*** L. | Summer | Panfilia Forest (FE) | 2 | 23/07/2022 |
| **5** | ***Hedera helix*** L. | Summer | Panfilia Forest (FE) | 2 | 23/07/2022 |
| **6** | *Populis alba* L. | Summer | Panfilia Forest (FE) | 2 | 23/07/2022 |
| **7** | ***Hedera helix*** L. | Summer | Panfilia Forest (FE) | 2 | 23/07/2022 |
| **8** | *Rubus caesus* L. | Summer | Panfilia Forest (FE) | 2 | 23/07/2022 |
| **9** | ***Hedera helix*** L. | Summer | Panfilia Forest (FE) | 2 | 23/07/2022 |
| **10** | *Rubus caesus* L. | Summer | Panfilia Forest (FE) | 2 | 23/07/2022 |
| **1** | *Carex pendula* Huds*.* | Summer | Panfilia Forest (FE) | 3 | 23/07/2022 |
| **2** | *Carex pendula* Huds*.* | Summer | Panfilia Forest (FE) | 3 | 23/07/2022 |
| **3** | *Cornus mas* L. | Summer | Panfilia Forest (FE) | 3 | 23/07/2022 |
| **4** | *Acer campestre* L. | Summer | Panfilia Forest (FE) | 3 | 23/07/2022 |
| **5** | *Rubus caesus* L. | Summer | Panfilia Forest (FE) | 3 | 23/07/2022 |
| **6** | *Sambucus nigra* L. | Summer | Panfilia Forest (FE) | 3 | 23/07/2022 |
| **7** | ***Hedera helix*** L. | Summer | Panfilia Forest (FE) | 3 | 23/07/2022 |
| **8** | *Rubus caesus* L. | Summer | Panfilia Forest (FE) | 3 | 23/07/2022 |
| **9** | ***Hedera helix*** L. | Summer | Panfilia Forest (FE) | 3 | 23/07/2022 |
| **10** | ***Hedera helix*** L. | Summer | Panfilia Forest (FE) | 3 | 23/07/2022 |
| **1** | ***Hedera helix*** L. | Autumn | Panfilia Forest (FE) | 1 | 02/11/2022 |
| **2** | ***Hedera helix*** L. | Autumn | Panfilia Forest (FE) | 1 | 02/11/2022 |
| **3** | *Rubus caesus* L. | Autumn | Panfilia Forest (FE) | 1 | 02/11/2022 |
| **4** | ***Hedera helix*** L. | Autumn | Panfilia Forest (FE) | 1 | 02/11/2022 |
| **5** | *Corylus avellana* L. | Autumn | Panfilia Forest (FE) | 1 | 02/11/2022 |
| **6** | ***Hedera helix*** L. | Autumn | Panfilia Forest (FE) | 1 | 02/11/2022 |
| **7** | *Carex pendula* Huds*.* | Autumn | Panfilia Forest (FE) | 1 | 02/11/2022 |
| **8** | *Carex pendula* Huds*.* | Autumn | Panfilia Forest (FE) | 1 | 02/11/2022 |
| **9** | *Rubus caesus* L. | Autumn | Panfilia Forest (FE) | 1 | 02/11/2022 |
| **10** | ***Hedera helix*** L. | Autumn | Panfilia Forest (FE) | 1 | 02/11/2022 |
| **1** | *Carex pendula* Huds*.* | Autumn | Panfilia Forest (FE) | 2 | 02/11/2022 |
| **2** | *Carex pendula* Huds*.* | Autumn | Panfilia Forest (FE) | 2 | 02/11/2022 |
| **3** | *Populus alba* L. | Autumn | Panfilia Forest (FE) | 2 | 02/11/2022 |
| **4** | *Carex pendula* Huds*.* | Autumn | Panfilia Forest (FE) | 2 | 02/11/2022 |
| **5** | *Rubus caesus* L. | Autumn | Panfilia Forest (FE) | 2 | 02/11/2022 |
| **6** | ***Hedera helix*** L. | Autumn | Panfilia Forest (FE) | 2 | 02/11/2022 |
| **7** | ***Hedera helix*** L. | Autumn | Panfilia Forest (FE) | 2 | 02/11/2022 |
| **8** | ***Hedera helix*** L. | Autumn | Panfilia Forest (FE) | 2 | 02/11/2022 |
| **9** | *Carex pendula* Huds*.* | Autumn | Panfilia Forest (FE) | 2 | 02/11/2022 |
| **10** | *Carex pendula* Huds*.* | Autumn | Panfilia Forest (FE) | 2 | 02/11/2022 |
| **1** | *Cornus mas* L. | Autumn | Panfilia Forest (FE) | 3 | 02/11/2022 |
| **2** | ***Hedera helix*** L. | Autumn | Panfilia Forest (FE) | 3 | 02/11/2022 |
| **3** | *Rubus caesus* L. | Autumn | Panfilia Forest (FE) | 3 | 02/11/2022 |
| **4** | ***Hedera helix*** L. | Autumn | Panfilia Forest (FE) | 3 | 02/11/2022 |
| **5** | *Sambucus nigra* L. | Autumn | Panfilia Forest (FE) | 3 | 02/11/2022 |
| **6** | ***Hedera helix*** L. | Autumn | Panfilia Forest (FE) | 3 | 02/11/2022 |
| **7** | *Rubus caesus* L. | Autumn | Panfilia Forest (FE) | 3 | 02/11/2022 |
| **8** | ***Hedera helix*** L. | Autumn | Panfilia Forest (FE) | 3 | 02/11/2022 |
| **9** | *Acer campestre* L. | Autumn | Panfilia Forest (FE) | 3 | 02/11/2022 |
| **10** | ***Hedera helix*** L. | Autumn | Panfilia Forest (FE) | 3 | 02/11/2022 |

Note: ^a^positive samples are in bold

**Figure S 1** Grid used for plant sample collection in the field. A 1 × 1 m grid with an approximately 20 cm pitch was centered on fruiting points (FPs) and used to sample the plants at its nodes surrounding the FP. The 10 plants closest to the FP were selected for each FP.

**Figure S 2** Alignment comparison between *Tuber magnatum* Picco Fluorescent *In Situ* Hybridization (FISH) specific probes and 18S sequences and its genetically closely related species. For each nucleotide position (column), polymorphisms with less than 50% nucleotide identity among sequences are shown; in contrast, conserved regions are shaded in grey. a) probe T.mag185, b) probe T.mag645, c) probe T.mag1313, d) probe T.mag1647. These alignments were generated using BioEdit 7.7.1 (Hall et al., 2011).

**Figure S 3** Maximum likelihood phylogeny of 18S gene sequences from *Tuber magnatum* Picco genetically close species used in this study to design *T. magnatum* FISH specific probes. The *Tuber* spp. used in the preliminary FISH probe specificity tests are indicated in bold. Accession numbers are indicated before each species name. Bootstrap values >75% are indicated at the nodes. Bar = 1 substitution every 100 positions.

**Figure S 4** Number of positive and negative collected plant samples in Spring 2023 from Città della Pieve (CDP), Panfilia Forest (PF) and Montefalcone nel Sannio (MNS), classified by families.

**Figure S 5** Co-hybridization FISH experiments on pure fungi. The evaluation was conducted using six different samples: *Tuber magnatum* Picco (A, 1) in the first column, *Tuber borchii* Vittad. (B, 2) in the second, *Tuber brumale* Vittad. (C, 3) in the third, *Tuber aestivum* Vittad. (D, 4) in the fourth, *Morchella eximia* Boud. (E, 5) as an outgroup species in the fifth, and *T. magnatum* as the negative control with water or AS-probes (F, 6) in the sixth. Co-hybridization experiments involved the generalist probe Euk516, targeting eukaryotic cells, which is visible in the second row of each panel (1-6) with a red signal. This was combined with one of the four newly designed probes (A-E), which emitted a green signal. For every co-detection, 2D images were captured from individual channels and displayed. The negative control was performed using either a nonsense probe (AS-Euk516) mixed with Euk516 or a hybridization buffer without any FISH probe, as shown in the rightmost column. The panels illustrate the following co-hybridizations: Panel I shows the co-hybridization with the *T. magnatum*-specific probe T.mag1647, Panel II with T.mag1313, Panel III with T.mag645, and Panel IV with T.mag185. The fluorochromes Atto565 and Atto633 were excited at 561 nm and 639 nm, respectively.

REFERENCES

Amicucci A, Zambonelli A, Giomaro G, Potenza L, Stocchi V (1998) Identification of ectomycorrhizal fungi of the genus *Tuber* by species-specific ITS primers. Mol Ecol 7:273-277.

ARPAE (2024). https://www.arpae.it (accessed 7 December 2024)

ARSARP (2024). https://www.arsarp.it (accessed 7 December 2024)

Baciarelli-Falini L, Rubini A, Riccioni C, Paolocci F (2006) Morphological and molecular analyses of ectomycorrhizal diversity in a man-made *T. melanosporum* plantation: description of novel truffle-like morphotypes. Mycorrhiza 16(7):475-484. https://doi.org/10.1007/s00572-006-0066-5

Blehert DS, Hicks AC, Behr M, Meteyer CU, Berlowski-Zier BM, Buckles EL, Coleman JTH, Darling RS, Gargas A, Niver R, Okoniewski JC, Rudd RJ, Stone WB (2009) Bat white-nose syndrome: an emerging fungal pathogen?. Science 323(5911):227-227. https://doi.org/10.1126/science.1163874

Gardes M, Bruns TD (1993) ITS primers with enhanced specificity for basidiomycetes-application to the identification of mycorrhizae and rusts. Mol Ecol 2(2):113-118. https://doi. org/10.1111/j.1365-294X.1993.tb00005.x

Gargas A, Trest MT, Christensen M, Volk TJ, Blehert DS (2009) *Geomyces destructans* sp. nov. associated with bat white-nose syndrome. Mycotaxon 108(1):147-154.

Hall T, Biosciences I, Carlsbad C (2011) BioEdit: an important software for molecular biology. GERF Bulletin of Biosciences 2:60-61.

Hansen K, Perry BA, Pfister DH (2005) Phylogenetic origins of two cleistothecial fungi, *Orbicula parietina* and *Lasiobolidium orbiculoides*, within the operculate discomycetes. Mycologia 97(5):1023-1033. https://doi.org/10.1080/15572536.2006.11832752

Hansen K, S Weber N, Landvik S (2008) Phylogenetic relationships and distribution of *Karstenella* (Pezizomycetes). Karstenia 48:13-19. http://urn.kb.se/resolve?urn=urn:nbn:se:nrm:diva-5053

ISTAT (2024). https://www.istat.it (accessed 7 December 2024)

Jeandroz S, Murat C, Wang Y, Bonfante P, Tacon, FL (2008) Molecular phylogeny and historical biogeography of the genus *Tuber*, the ‘true truffles’. J Biogeogr 35(5):815-829. https://doi.org/10.1111/j.1365-2699.2007.01851.x

Kang HJ, Sigler L, Lee J, Gibas CFC, Yun SH, Lee YW (2010) *Xylogone ganodermophthora* sp. nov., an ascomycetous pathogen causing yellow rot on cultivated mushroom *Ganoderma lucidum* in Korea. Mycologia 102(5):1167-1184. https://doi.org/10.3852/09-304

Landvik S (1994) Relationships of the genus *Glaziella* (Ascomycota) inferred from 18S rDNA sequences. Syst Ascomycetum 13:13-23.

Nasr S, Bien S, Soudi MR, Alimadadi N, Shahzadeh Fazeli SA, Damm U (2018) Novel *Collophorina* and *Coniochaeta* species from *Euphorbia polycaulis*, an endemic plant in Iran. Mycol Prog 17:755-771. https://doi.org/10.1007/s11557-018-1382-9

NCBI (2024). https://www.ncbi.nlm.nih.gov (accessed 8 December 2024)

O'Donnell K, Cigelnik E, Weber NS, Trappe JM (1997) Phylogenetic relationships among ascomycetous truffles and the true and false morels inferred from 18S and 28S ribosomal DNA sequence analysis. Mycologia 89(1):48-65. https://doi.org/10.1080/00275514.1997.12026754

Percudani R, Trevisi A, Zambonelli A, Ottonello S (1999) Molecular phylogeny of truffles (Pezizales: Terfeziaceae, Tuberaceae) derived from nuclear rDNA sequence analysis. Mol Phylogenet Evol 13(1):169-180. https://doi.org/10.1006/mpev.1999.0638

REGIONE EMILIA ROMAGNA (2024). https://ambiente.regione.emilia-romagna.it/it (accessed 7 December 2024)

REGIONE UMBRIA (2024). https://www.umbriageo.regione.umbria.it (accessed 7 December 2024)

Ruibal C, Gueidan C, Selbmann L, Gorbushina AA, Crous PW, Groenewald JZ, Muggia L, Grube M, Isola D, Schoch CL, Staley JT, Lutzoni F, De Hoog GS (2009) Phylogeny of rock-inhabiting fungi related to Dothideomycetes. Stud Mycol 64(1):123-133. https://doi.org/10.3114/sim.2009.64.06

Selbmann L, De Hoog GS, Mazzaglia A, Friedmann EI, Onofri S (2005) Fungi at the edge of life: cryptoendolithic black fungi from Antarctic desert. Stud Mycol 51(1):1-32. https://doi.org/10.3114/sim.2009.64.06

Spatafora JW, Sung GH, Johnson D, Hesse C, O’Rourke B, Serdani M ... Schoch CL (2006) A five-gene phylogeny of Pezizomycotina. Mycologia 98(6):1018-1028. https://doi.org/10.1080/15572536.2006.11832630

Suh SO, Marshall CJ, Mchugh JV, Blackwell M (2003) Wood ingestion by passalid beetles in the presence of xylose‐fermenting gut yeasts. Mol Ecol 12(11):3137-3145. https://doi.org/10.1046/j.1365-294X.2003.01973.x

Suh SO, McHugh JV, Pollock DD, Blackwell M (2005) The beetle gut: a hyperdiverse source of novel yeasts. Mycol Res 109(3):261-265. https://doi.org/10.1017/S0953756205002388

Tanabe Y, Nagahama T, Saikawa M, Sugiyama J (1999) Phylogenetic relationship of *Cephaliophora* to nematophagous hyphomycetes including taxonomic and nomenclatural emendations of the genus *Lecophagus*. Mycologia 91(5):830-835. https://doi.org/10.1080/00275514.1999.12061088

White TJ, Bruns T, Lee S, Taylor J (1990) Amplification and direct sequencing of fungal ribosomal RNA genes for phylogenetics. In: Innis MA, Gelfand DH, Sninsky JJ, White TJ (eds) PCR protocols: a guide to methods and applications, Academic Press, Inc., New York, NY, USA, 18 pp 315–322.
